# Adaptively partitioned block-based contrast enhancement and its application to low light-level video surveillance

**DOI:** 10.1186/s40064-015-1226-x

**Published:** 2015-08-19

**Authors:** Seungwon Lee, Nahyun Kim, Joonki Paik

**Affiliations:** Chung-Ang University, 221 Heukseok-Dong, Dongjak-Gu, Seoul, 156-756 Korea

**Keywords:** Image enhancement, Contrast enhancement, Backlighting compensation, Guided filter

## Abstract

This paper presents a dark region detection and enhancement method with low computational complexity for low-cost imaging devices. Conventional contrast enhancement methods generally have an oversaturation problem while brightness of the dark region increases. To solve this problem, the proposed method first divides an input image into dark object and bright background regions using adaptively partitioned blocks. Next, the contrast stretching is performed only in the dark region. The major advantage of the proposed method is the minimized block artifacts using optimally partitioned blocks using fuzzy logic and a refining step to accurately detect boundaries between two regions. Experimental results show that the proposed method can efficiently enhance the contrast of backlit images without the oversaturation problem. Because of low computational complexity, the proposed method can be applied to enhance very low light-level video sequences for video surveillance systems.

## Background

Recent advances in digital image processing systems enable users to easily acquire high quality images using compact, inexpensive digital cameras. However, a limited dynamic range is still a bottleneck of the camera technology (Debevec and Malik 1997). Because of the limited dynamic range, an image having both dark objects and bright background either loses object information or becomes over-saturated in the background region. An efficient image enhancement algorithm is required to enhance the contrast of the dark objects without over-saturation in the background.

Histogram equalization (HE) is a global contrast enhancement method for solving the unbalanced illumination problem in the image (Wang and Ye 2005). However, it tends to make the background saturated and amplifies the noise in the dark region of the image. For addressing this issue, several versions of improved HE algorithms have been proposed. The adaptive histogram equalization (AHE) method adaptively partitions the image into multiple sub-blocks for block-based local histogram equalization at the cost of blocking artifacts (Zimmerman et al. 1998). The bi-histogram equalization (BHE) method enhances the contrast of backlit images while preserving the average brightness. It is difficult to accurately separate background and object regions using a single threshold value to bisect the histogram (Kim 1997). The dualistic sub-image histogram equalization (DSIHE) method is similar to BHE except that the threshold value is selected using the median value of an image (Wan et al. 2003). As a result, DSIHE enhances the contrast of the images while preserving the mean brightness. However, the disordered histogram results in either over-saturation or under exposure. The recursive mean-separate histogram equalization (RMSHE) method performs iterative BHE for preserving the average brightness (Chen and Ramlli 1999). However, the effect of contrast enhancement decreases as the iteration continues. The gain controllable clipped histogram equalization (GC-CHE) method dynamically controls the clipping level of the histogram for appropriately re-distributing the dynamic range (Kim and Paik 2008).

For solving the problem of above mentioned global contrast enhancement methods, locally adaptive contrast enhancement methods have also been proposed. Kim et al. divided backlit and background regions using a set of optimal threshold values. Contrast enhancement is then performed only in the backlit region (Kim et al. 2013). However, blocking artifacts are generated in the boundary between the two regions. The retinex-based method can be considered as a locally adaptive contrast enhancement method that reduces the illumination dependency and stretches the dynamic range of only reflectance component using a Gaussian filter (Kim et al. 2011). However, if the size of the Gaussian filter is not appropriately selected, the processed image contains halo effect and color distortion.

**Fig. 1:**
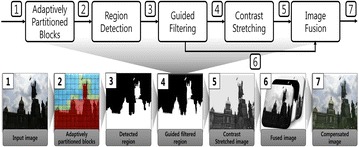
Block diagram of the proposed dark region detection and enhancement (*top*) and the corresponding output of each block (*bottom*)

In this context most conventional contrast enhancement methods have the problem of under- or over-saturation with color distortion. To overcome this problem, adaptively partitioned block-based dark region detection and enhancement is presented as shown in Fig. 1.

As show in Fig. 1, the proposed method separates the image into the dark and background regions using adaptively partitioned blocks based on the optimal threshold value computed by fuzzy C-means clustering (FCM). More specifically, the proposed method partitions the input image into non-overlapped blocks of size $64\times 64$, and classifies them as dark, background, and ambiguous regions using the optimal threshold. The ambiguous blocks are further partitioned into four sub-blocks, which are then re-classified in the same manner. This partitioning process is repeated until the size of a block becomes $4\times 4$. Finally, the detected block-based dark region is refined using the guided filter for removing block artifacts in the enhanced image region (He et al. 2010). The filtered dark region is enhanced by contrast stretching, and the final output is obtained by fusing the enhance dark and input background regions.

This paper is organized as follows. “Adaptively partitioned block-based dark region detection and enhancement” section presents the automatic object segmentation algorithm, and “Experimental results” section presents experimental results, and “Conclusion” section concludes the paper.

## Adaptively partitioned block-based dark region detection and enhancement

Because of the limited dynamic range of a digital camera, many consumers photographs are subject to backlit image degradation. The backlit image has a bi-modal histogram where one mode corresponds to the dark range and the other to the bright range in the background region.

**Fig. 2:**
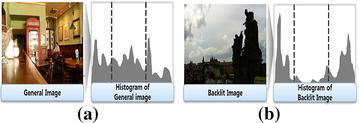
Comparison of a normal and backlit images with their histograms: **a** a normally illuminated image and, **b** a backlit image

Figure 2 compares histograms of a normally illuminated and backlit images. The normal image has evenly distributed intensity value as shown in Fig. 2a, while the backlit image has a two-mode histogram as shown in Fig. 2b. We can also observe that bright background region gives more visual information than the dark backlit region that has narrow dynamic range of low intensities.

In order to selectively enhance the contrast with over-saturation problem, the dark backlit region is accurately detected using adaptively partitioned blocks. Contrast of the backlit region is then enhanced while preserving the brightness of the background region.

### Adaptive backlit region detection

If the backlit region is segmented in the pixel level using a threshold of brightness, the low intensity in the background region is misclassified as the backlit region, and the high intensity in the backlit region is misclassified as the background region. This sub section presents the fast backlit region detection algorithm using adaptively partitioned blocks while minimizing the misclassification. Because the degree of brightness in the backlit region varies by image, an optimal threshold for detecting the backlit region has to be adaptively selected. Under assumption that the image can be divided into the background and backlit regions, the optimal threshold value is selected using a clustering method. Although the k-means clustering method is widely used for pattern classification because of its simplicity and robustness to noise, it is not suitable for region classification if an image contains ambiguous regions. To overcome the limitation of the k-means clustering algorithm, the proposed method uses the fuzzy C-mean (FCM) clustering method that estimates the distribution of the brightness in the image to adaptively select the optimal threshold value (Shen et al. 2005). In this work the number of clusters in the FCM algorithm is set to two for backlit and background regions. The optimal means of the two clusters are defined as

1 $$\begin{aligned} {J_{FCM}} = \sum \limits _{i = 1}^N {\sum \limits _{j = 1}^C {{{( {{u_{ij}}}) }^m}{{\left\| {{x_i} - {c_j}} \right\| }^2}} }, \end{aligned}$$

where $x_{i}$ represents the brightness of the *i*th pixel in the image, $c_{j}$ the mean of the *j*th cluster, *N* the total number of pixels in the image, *C* the number of clusters, *m* a weighting exponent on each fuzzy membership, and $u_{ij}$ the degree of $x_{i}$ contained in the *i*th cluster. In the experiment, $C=2$, and $m=2$ were used. To reduce the computational complexity, the input image is divided into non-overlapped $64\times 64$ blocks. $x_{i}$ is then used as the mean of the brightness of each non-overlapped block. The optimal thresholds $c_{1}$ and $c_{2}$ for dark and background regions, respectively, can be selected by minimizing (1).

**Fig. 3:**
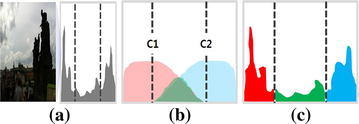
The optimal thresholds decision using FCM: **a** an input image and its histogram,** b** concept of partitioning the three regions using two optimal thresholds $c_1$ and $c_2$,** c** result of histogram (*red* dark region,* green* ambiguous region,* blue* bright region)

Figure 3 shows the optimal threshold decision process using FCM. Figure 3a shows an input backlit image and its histogram. The intensity distribution is concentrated in both dark and bright ranges. The histogram of the input image is segmentation by the optimal thresholds $$c_1 $$ and $$c_2 $$ as shown in Fig. 3b. If an intensity value is less than $$c_1$$, the corresponding pixel is classified as the dark backlit region. On the other hand, if an intensity value is larger than $$c_2 $$, the corresponding pixel is classified as the bright background region. Figure 3c shows the result of the histogram segmentation of Fig. 3a using FCM.

The proposed backlit region detection partitions the input image into non-overlapped blocks of size $64\times 64$ and classifies each block as one of dark, background, and bright regions using the optimal thresholds. The classification of the blocks is determined as

2 $$ L_{{k,l}}  = \left\{ {\begin{array}{*{20}ll}    {2,} & {b_{{\max}} ^{{{{k,l}} }}  \le c_{1} \;or,\;\frac{{D_{{k,l}} }}{{N_{k} }} > 0.75}  \\    {1,} & {c_{2}  \le b_{{\min}} ^{{{{k,l}} }} }  \\    {0,} & {otherwise}  \\   \end{array} } \right., $$

where *k* represents the step of the partitioning blocks, and *l* the block number in the *k*th step. $L=2$ corresponds to a dark block, 1 to a bright block, and 0 to an ambiguous block. $b_{max}$ and $b_{min},$ respectively represent the maximum and minimum brightness values in the corresponding block. $N_{k}$ represents the total number of pixels in the block at the *k*th step. $D_{k,l}$ represents the number of pixels smaller than $c_{1}$ in the *l*th block, and $\frac{{{D_{k,l}}}}{{{N_k}}}$ the ratio of the dark pixels in the block, which the misclassification caused by illumination change, noise, and small bright colors in the dark region. The classified ambiguous blocks are further partitioned into four sub-blocks, which are then re-classified in the same manner. The hierarchical partitioning repeats until the block size becomes $4\times 4$.

The guided filter is used to refine the boundary of the dark region while preserving the edge of the original image (Paris et al. 2008). The guidance image *I* is used to filter a guided image *L* that has block-wise partitioned dark and background regions. Let $I_{x}$ and $L_{x}$ be the $1\times 3$ color vector of the guidance image at pixel *x* and the corresponding label, and $w_{k}$ be the kernel window centered at *x*, then the guided filter is formulated as

3 $$\begin{aligned} {G_x} = \mathrm{{a}}_x^\mathrm{{T}}{\mathrm{{I}}_x} + {b_x}, \end{aligned}$$

where $a_{x}$ is a $3\times 1$ coefficient vector, and $b_x$ is a scalar defined as

4 $$\begin{aligned} {\mathrm{{a}}_x} = {\left(\sum \limits _x + \varepsilon U\right)^{ - 1}}\left(\frac{1}{{\left| \omega \right| }}\sum \limits _{i \in {\omega _x}} {{\mathrm{{I}}_i}{G_i} - {\mu _x}{{\bar{G}}_x}}\right), \end{aligned}$$

5 $$\begin{aligned} {b_x} = {\bar{G}_x} - \mathrm{{a}}_x^\mathrm{{T}}{\mu _x}, \end{aligned}$$

where $\mu _{x}$ represents the mean of $I_x$ in $\omega _{x}$, $\left| {{\omega _x}} \right|$ the number of pixels in $\omega _{x}$, ${\bar{G}_x} = \frac{1}{{\left| \omega \right| }}\sum \nolimits _{i \in {\omega _x}} {{G_i}}$ the mean of *G* in $\omega _{x}$. $\sum \nolimits _x$ the $3\times 3$ covariance matrix of $I_x$ in $\omega _{x}$, and *U* the $3\times 3$ identity matrix. The degree of smoothing of the guided filter is adjusted by parameter $\varepsilon$. The larger $\varepsilon$ is, the smoother the filtered image becomes.

**Fig. 4:**
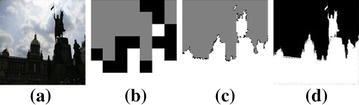
Results of the proposed adaptively partitioned block-based dark region detection method. (*Black* ambiguous regions,* white* dark region, and* gray* background region):** a** input image,** b** $k=1\,({n=64})$,** c** $k=5\,( {n=4})$, and** d** the finally refined dark region

The detected dark region using the adaptively partitioned blocks is shown in Fig. 4. The original image has obvious contrast of the brightness between the backlit and background regions as shown Fig. 4a. Figure 4b and c, respectively show the first and last steps of hierarchical block partitioning. In case of *k* = 5, the detected backlit region looks almost the same to the actual backlit region, but there still remains blocking artifacts and ambiguous regions at the boundary between backlit and background regions. The image enhancement with the this coarsely segmented region occurs unnatural boundary and halo effects. In order to reduce the blocking artifacts shown in Fig. 3c, the guided filter is used to generate a continuous-valued weighting map for the ambiguous region. Figure 3d shows the result of the guided filter applied to the fifth step of partitioned blocks to generate a naturally looking dark region.

### Contrast enhancement of the dark region

If a global contrast stretching method is used to enhance objects in the dark region, the bright background is over-saturated. To solve this problem, contrast stretching is performed only for the detected dark region. The traditional contrast stretching method changes the brightness of the entire image using the minimum and maximum intensity values as

6 $$\begin{aligned} {\hat{I}_x} = \frac{{{I_x} - {I_{\min }}}}{{{I_{\max }} - {I_{\min }}}} \times 255, \end{aligned}$$

where $I_{min}$ and $I_{max}$ respectively represent the minimum and maximum intensity value of the image (Gonzalez and Woods 2008). For this reason, the bright background region is over-saturated by increasing the intensity of the dark region. To avoid this problem, the contrast stretching ends-in search (CSES) method has been proposed in Srinivasan and Balram (2006), which uses user-selected thresholds $I_{min}$ and $I_{max}$. Although CSES can efficiently enhance the contrast for the desired region, the background region is still over-saturated. On the other hand, the proposed method adaptively selects the thresholds, and performs contrast stretching in the refined dark region using a guided filter.

The proposed contrast stretching method can be formulated as

7 $$\begin{aligned} {\hat{I}_x} = \frac{{{I_x} - {I_{\min }}}}{{{c_1} - {I_{\min }}}} \times {c_2}, \end{aligned}$$

where $c_{1}$ and $c_{2}$ represent the adaptively chosen minimum and maximum thresholds, respectively. Since a backlit image consists of both dark and bright regions, the proposed method considers only low brightness pixels in the dark region. $c_{1}$ and $c_{2}$ determine if the corresponding pixel falls into either dark or background region. Simple multiplication of an appropriate constant and a low intensity value in the background region results in unnatural boundary between dark and background regions. On the other hand, multiplication by $c_{2}$ prevents dark pixels from becoming excessively bright, and thus reduces unnatural boundary artifacts. The efficient contrast stretching is performed using adaptive thresholds estimated by FCM. The finally enhanced image is created by the adaptive combination of contrast enhanced and the original images using the refined dark region as

8 $${E_x} = {G_x} \times \hat{I_x} + (1 - {G_x}) \times {I_x},$$

where ${G_x}$ represents the refined dark region, ${\hat{I}_x}$ the result of contrast stretching, and ${I_x}$ the input image. The finally enhanced image is generated by the combination of the enhanced image by (7) and the original image using the ratio of $G_{x}$, which makes the boundary smooth.

**Fig. 5:**
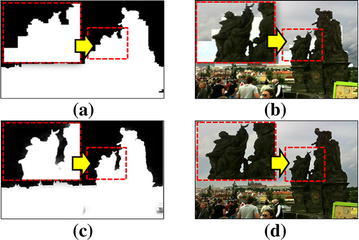
Results of the proposed adaptively partitioned block-based dark region detection method block-partitioning results of **a** the first step [$k=1\,( {n=64})$] and **b** the fifth step [$k=5\,( {n=4})$]

Figure 5 shows results of the enhancement of the proposed dark region detection method using a guided filter. The boundary between the dark and background regions is blocky as shown in 5a. The boundary of the object in the backlit region using the guided filter is well-refined as shown in Fig. 5b. The result of the enhancement of the backlit image smoothly changes the brightness at the boundary between the dark and background regions.

## Experimental results

This section presents experimental results of the proposed adaptively partitioned block-based contrast enhancement method. The proposed enhancement method first transforms the input color image into the hue-saturation-value (HSV) color space, and performs contrast enhancement for only V channel, which contains brightness information. To evaluate the effectiveness of the proposed method, this paper compared the proposed method with gain-controllable clipped histogram equalization (GC-CHE) (Kim and Paik 2008) and multi scale Retinex with color restoration (MSRCR) method (Jobson et al. 1997). The MSRCR method restores the color contrast by applying the conventional Retinex method to each RGB channel.

**Fig. 6:**
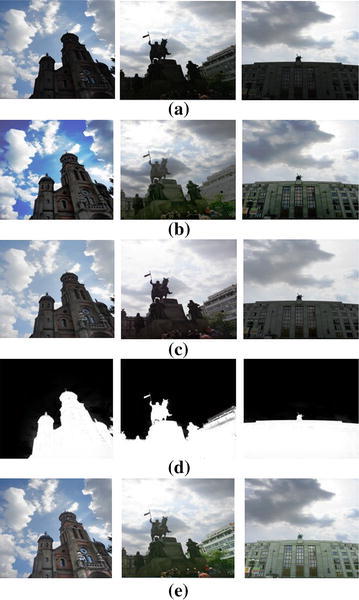
Results of four different contrast enhancement methods: **a** input image,** b** GC-CHE,** c** MSRCR,** d** alpha map of guided filter based backlit region, and** e** the proposed method

Figure 6 compares the performance of the proposed method with conventional contrast enhancement methods. The original image as shown in Fig. 6a lost the information of the objects in the dark backlit region. The result of GC-CHE overcame the over-saturation problem in the background region such as the sky as shown in Fig. 6b. However, it cannot significantly enhance the backlit region. Figure 6c is the result of MSRCR, which enhances the detail of objects in the backlit region better than the other two methods. However, MSRCR still exhibits color distortion and over-saturation. Figure 6e shows the enhancement result of the proposed method. The detail of objects were better restored than existing methods. Since it performs the enhancement only in the detected dark region as shown in Fig. 6d.

A low light video containing a vehicle head lamp or streetlamps has a bi-modal histogram similar to the backlit image by the sum. Therefore, the proposed method can also be used to enhance such low light video sequences.

**Fig. 7:**
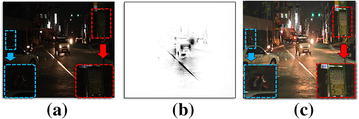
Result of the proposed contrast enhancement method for a low light video: **a** input image frame, **b** detected highlighted region, and **c** result of the proposed enhancement method for the low light image

Figure 7 shows the result of the proposed enhancement method for a low light image with the highlighted region in the center. Figure 7a is a low light image acquired by a video camera. The background region becomes darker because of vehicle lamps makes the bi-modal histogram like the sun in a backlit image. Figure 7b shows the detected highlighted region, where the block area which represents the bright lamps. Figure 7c shows the result of contrast enhancement using detected highlighted region shown in Fig. 7b. The detail of the objects in dark regions is better restored than the input image without over-saturation or color distortion in the highlighted region.

**Fig. 8:**
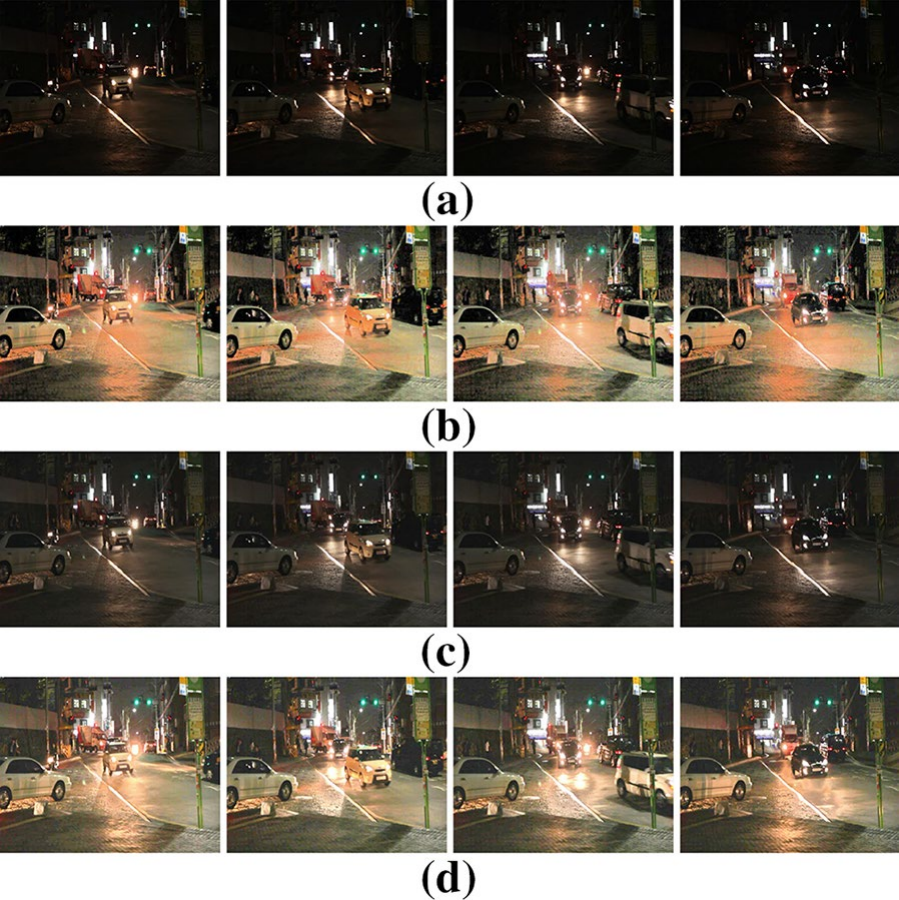
Comparison of various contrast enhancement methods for a low light video. Four frames of **a** an input low light video,** b** enhance results using HE,** c** MSRCR, and** d** the proposed method

Figure 8 compares the performance of the proposed method with the HE and MSRCR methods. The enhanced image using HE can restore the detail of objects in the backlit region at the cost of over-saturation in the highlighted region as shown in Fig. 8b. On the other hand, MSRCR enhances the detail of the objects in the backlit region better than the HE at the cost insufficient enhancement in the backlit region as shown in Fig. 8c. As shown in Fig. 8d, the proposed method can enhance the contrast in both dark and bright regions without only color distortion problems.

For objective evaluation of the performance of contrast enhancement methods, average entropy (AE) is computed as

9 $$\begin{aligned} {AE} = \frac{1}{N}\sum \limits _{k = 0}^{255} {P_{out}(X_k)} \times {log_{2} P_{out}(X_k)}, \end{aligned}$$

where $P_{out}(X_k)$ is the normalized probability of the *k*th gray level, and *N* the number of pixels in the image.

**Table 1:**
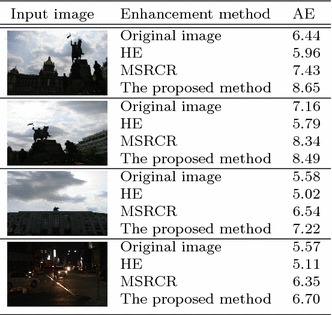
Quantitative evaluating using AE

Table 1 shows AE value of various contrast enhancement methods on the set of three test images. The higher AE value indicates that more detail of the image is restored by the enhancement method. According to Table 1, the proposed method produces higher AE value than any other conventional methods.

The gain-controlled clipped histogram equalization (GC-CHE) method (Kim and Paik 2008) is compared with the proposed method since it is known as the best histogram modification approach in enhancing the contrast of digital images. Although various improved or modified versions were proposed in the literature, the original work of Kim and Paik (2008) is the best candidate of the performance comparison without significantly increasing the computational load. The multi-scale retinex method (Jobson et al. 1997) is also known as the first work or retinex theory-based contrast enhancement method. Although various different versions of retinex-based methods were proposed in the literature, the original work of Jobson et al. (1997) is the most appropriate for performance comparison with similar amount of computational load.

In order to demonstrate the performance of the proposed method, we first used subjective comparison as shown in Fig. 8. The input low-light video frames shown in Fig. 8a is suitable to evaluate the performance of contrast enhancement since it contains both dark and saturated regions. In addition to subjective comparison, we evaluated average entropy to represent how evenly the brightness is spread in the processed image.

## Conclusion

This paper has presented a method to enhance the contrast of two-mode brightness image. Conventional contrast enhancement methods have over-saturation and color distortion problems. To solve these problems, the proposed method divides the image into dark and background regions using adaptively partitioned blocks by two optimal threshold values computed by fuzzy C-means clustering in the V channel of the HSV color space. The proposed contrast stretching process is performed only in the detected dark region. The major advantage of the proposed method is the minimized block artifacts due to adaptively partitioning the image according to the optimal threshold and the refining step to detect the dark regions. The proposed method automatically segments backlit region and the background region. It does not need manual seed region selection for segmentation and has low segmentation complexity than heavy segmentation methods such as graph cut-based method. Experimental results showed that the proposed method can better enhance the contrast than existing methods in the sense of both minimizing over-saturation in the bright background region and preserving details in the dark region.
